# Supplementary material for: The brain-derived neurotrophic factor Val66Met polymorphism increases segregation of structural correlation networks in healthy adult brains
Source: PeerJ. 2020 Aug 5;8:e9632. doi: 10.7717/peerj.9632 (PMC7414771; doi:10.7717/peerj.9632)
Supplement: Supplemental Information 2 [file peerj-08-9632-s002.pdf]

Supplementary Analysis 1: a negative-edge-removed version analysis

Purpose:

For network construction in the main analysis, we defined anatomical connections as statistical associations in cortical thickness between the brain regions in order to characterize human brain networks. We evaluated statistical similarities in cortical thickness between all region pairs using Spearman correlation coefficients (*r*) across subjects in each group. The correlation coefficient *r* ranges in value from 1 (positive correlation) and −1 (negative correlation), and the closer to 1 or −1 the more significant the correlation.

To avoid the complication of statistical feature descriptions in subsequent graph theoretical analyses, we converted *r* values into absolute values in the main analysis. However, the biological basis of the the correlation between two regions is still not clear, and it is difficult to determine a better way to treat the negative correlation. To confirm that our main analysis results were not distorted by treating connections with opposite (i.e., negative) directions as equivalent to connections with the same (i.e., positive) direction, we conducted this supplementary analysis.

Result:

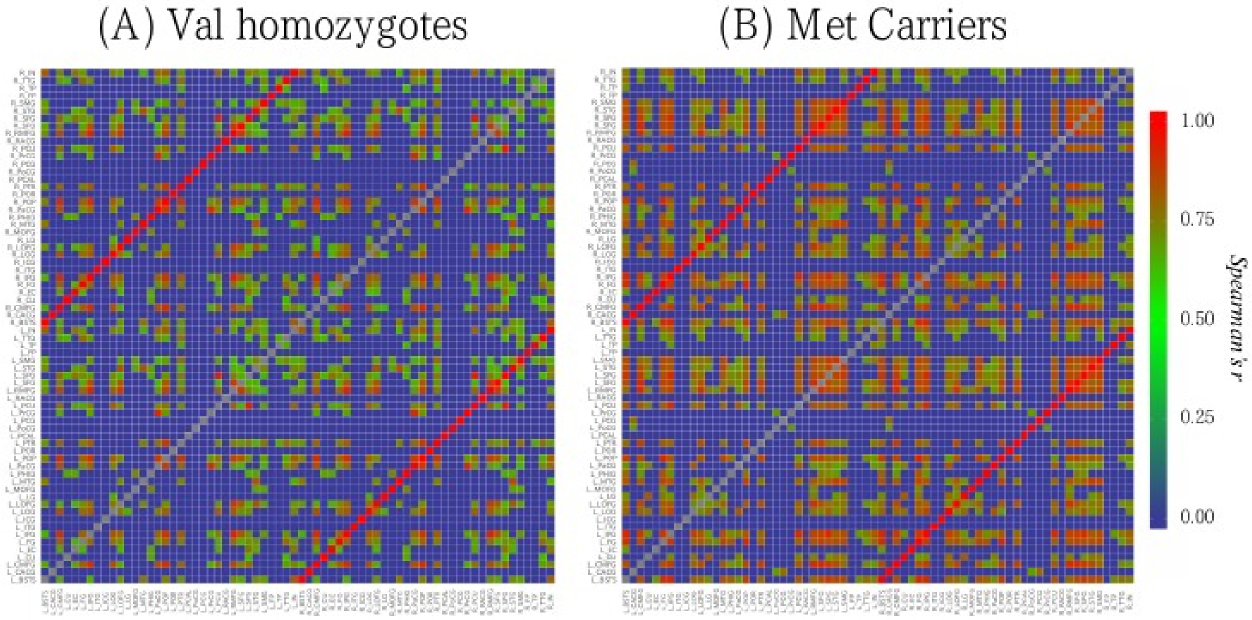

Figure 1. Heat maps of the brain structural correlation network of the BDNF Val homozygotes (A) and Met carriers (B). These heat maps represent each strength of inter-regional cortical thickness correlation of 68 brain regions. The color scale corresponds to the absolute values of Spearman's correlation coefficient. In a heat map of the brain network, grids represent sub-divided brain regions (i.e., modules). From these grid patterns, we observed that the Met carrier brain network had clearer module architecture than the Val homozygote brain network. When we focused on colors in each heat map, the Met carriers' map had more red areas whereas the Val homozygote heat map had more green areas. This observation suggested that the Val homozygote networks consisted of middle connections whereas the Met carrier networks had more connections with high strength. In both groups, each brain region had the strong correlation with the counterpart of the region; these correlations are described in the heat maps as the red lines running diagonally. The abbreviations of the brain regions used in these heat maps are summarized in Supplementary Table 1.

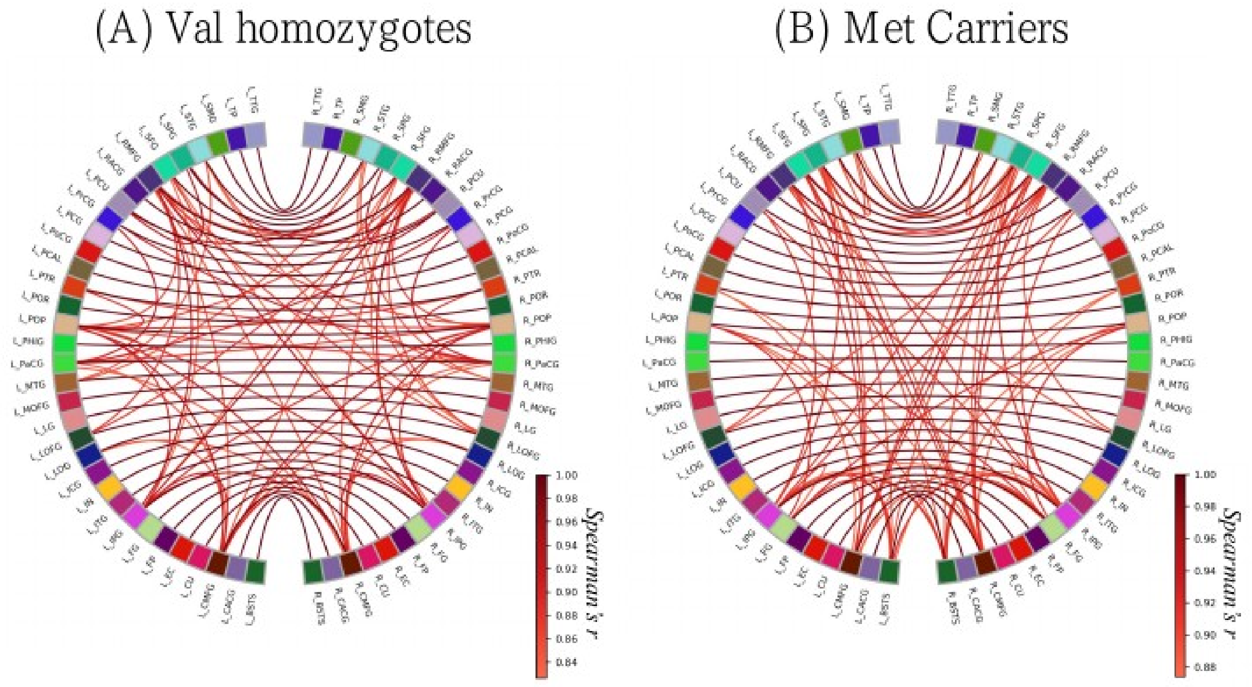

Figure 2. Circular graphs of brain structural correlation network of the BDNF Val homozygotes (A) and Met carriers (B). These circular graphs are describing the inter-regional cortical thickness correlation, where the nodes represent brain regions, and the edges represent undirected connections between the regions. Each of the edge weight corresponds to the absolute value of Spearman's correlation coefficient. In these circular graphs, we only showed the 100 strongest connections. The Met carrier network had fewer brain regions related to the 100 strongest connections than the Val homozygote network. The abbreviations of the brain regions used in these circular graphs are summarized in Supplementary Table 1.

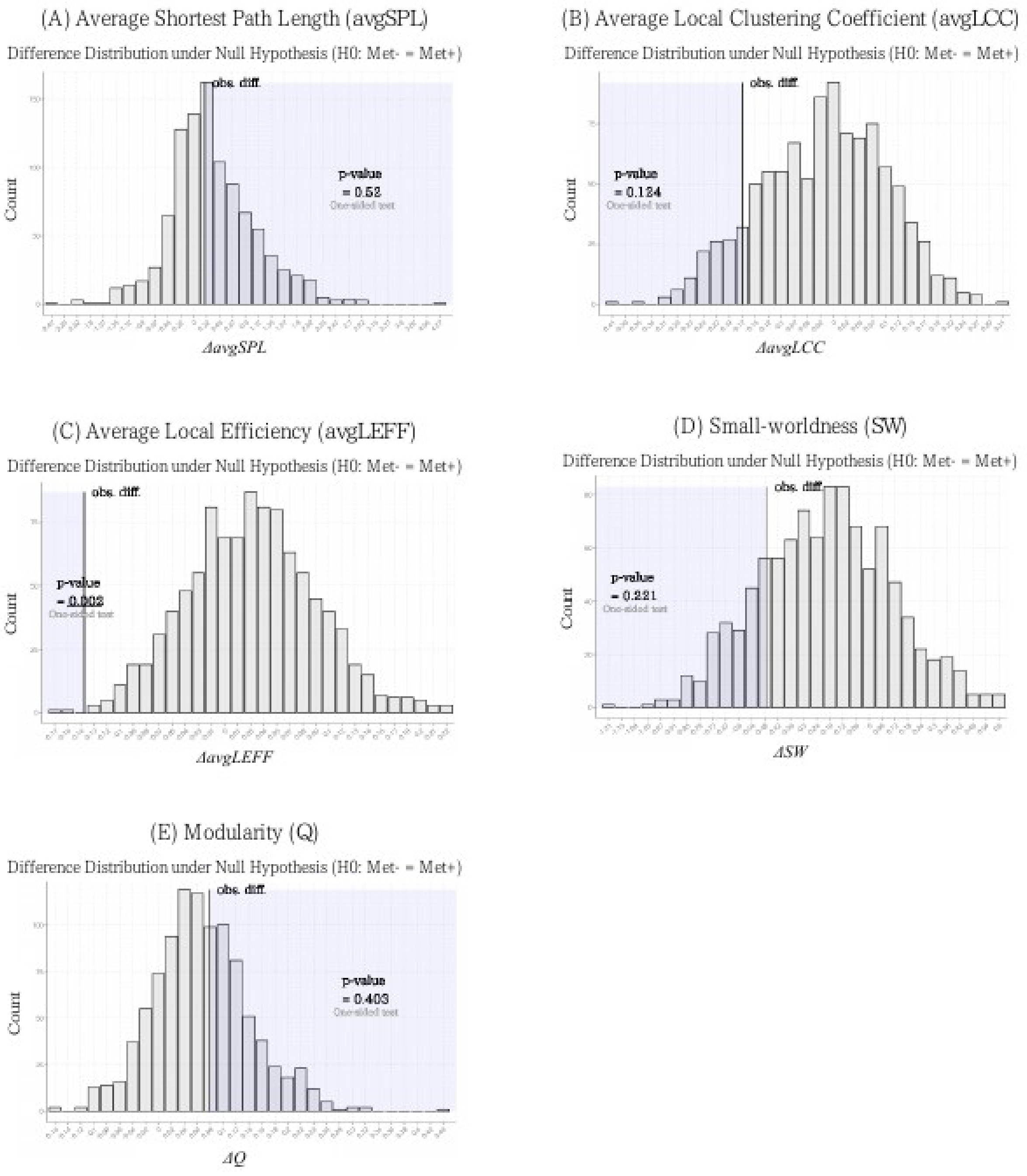

Figure 3. Permutation distributions and p-values of the network measures calculated from the structural correlation networks. These histograms were obtained by repeating the calculation of the network measure difference between the randomly-partitioned groups of subjects. Each of the p-values was calculated as the frequency that the simulated differences were greater (or less) than each of the observed differences. The average local efficiency (*avgLEFF*) of the Met carriers' network was larger than that of the Val homozygotes' network, and the statistical difference between the *avgLEFF* between the two networks was

significant (p-value = 0.002) (C). There were no significant differences between the two groups in regard to the average shortest path length (*avgSPL*), the average local clustering coefficient (*avgLCC*), small-worldness (*SW*), and modularity (*Q*) (p-value $\geqq$ 0.01) (A, B, D, and E).

Weighted Global Network Measure	Val Homozygotes	Met Carriers	p-value
Average Shortest Path Length (*avgSPL*)	1.873	1.663	0.520
Average Local Clustering Coefficient (*avgLCC*)	0.553	0.715	0.124
Average Local Efficiency (*avgLEFF*)	0.491	0.627	**0.002** *
Small-worldness (*SW*)	0.990	1.438	0.221
Modularity (*Q*)	0.201	0.120	0.403

Table. The statistical comparison result regarding network measures of the brain structural correlation networks from the BDNF Val homozygotes and Met carriers. The bold text with '*' indicates significance (p-value < 0.01). Abbreviations: BDNF = brain-derived neurotrophic factor, Val = valine, Met = methionine.

Conclusion:

The results of this supplementary analysis with the pre-processing to remove the negative correlation were similar to the main analysis. This suggests that treating negative correlations the same as positive correlations did not affect the conclusions of this study main analysis.
