# Supplementary material for: The brain-derived neurotrophic factor Val66Met polymorphism increases segregation of structural correlation networks in healthy adult brains
Source: PeerJ. 2020 Aug 5;8:e9632. doi: 10.7717/peerj.9632 (PMC7414771; doi:10.7717/peerj.9632)
Supplement: Supplementary file 27 [file peerj-08-9632-s003.odt]

|  | FreeSufer-defined Region Name |  | Abbreviation |  |  |  | FreeSufer-defined Region Nam |  | Abbreviation |  |
| --- | --- | --- | --- | --- | --- | --- | --- | --- | --- | --- |
|  | bankssts-l(r)h |  | L(R)_BSTS |  |  |  | parahippocampal-l(r)h |  | L(R)_PHIG |  |
|  | caudalanteriorcingulate-l(r)h |  | L(R)_CACG |  |  |  | parsopercularis-l(r)h |  | L(R)_POP |  |
|  | caudalmiddlefrontal-l(r)h |  | L(R)_CMFG |  |  |  | parsorbitalis-l(r)h |  | L(R)_POR |  |
|  | cuneus-l(r)h |  | L(R)_CU |  |  |  | parstriangularis-l(r)h |  | L(R)_PTR |  |
|  | entorhinal-l(r)h |  | L(R)_EC |  |  |  | pericalcarine-l(r)h |  | L(R)_PCAL |  |
|  | frontalpole-l(r)h |  | L(R)_FP |  |  |  | postcentral-l(r)h |  | L(R)_PoCG |  |
|  | fusiform-l(r)h |  | L(R)_FG |  |  |  | posteriorcingulate-l(r)h |  | L(R)_PCG |  |
|  | inferiorparietal-l(r)h |  | L(R)_IPG |  |  |  | precentral-l(r)h |  | L(R)_PrCG |  |
|  | inferiortemporal-l(r)h |  | L(R)_ITG |  |  |  | precuneus-l(r)h |  | L(R)_PCU |  |
|  | insula-l(r)h |  | L(R)_IN |  |  |  | rostralanteriorcingulate-l(r)h |  | L(R)_RACG |  |
|  | isthmuscingulate-lh |  | L(R)_ICG |  |  |  | rostralmiddlefrontal-l(r)h |  | L(R)_RMFG |  |
|  | lateraloccipital-l(r)h |  | L(R)_LOG |  |  |  | superiorfrontal-l(r)h |  | L(R)_SFG |  |
|  | lateralorbitofrontal-l(r)h |  | L(R)_LOFG |  |  |  | superiorparietal-l(r)h |  | L(R)_SPG |  |
|  | lingual-l(r)h |  | L(R)_LG |  |  |  | superiortemporal-l(r)h |  | L(R)_STG |  |
|  | medialorbitofrontal-l(r)h |  | L(R)_MOFG |  |  |  | supramarginal-l(r)h |  | L(R)_SMG |  |
|  | middletemporal-l(r)h |  | L(R)_MTG |  |  |  | temporalpole-l(r)h |  | L(R)_TP |  |
|  | paracentral-l(r)h |  | L(R)_PaCG |  |  |  | transversetemporal-l(r)h |  | L(R)_TTG |  |
