# Supplementary material for: The brain-derived neurotrophic factor Val66Met polymorphism increases segregation of structural correlation networks in healthy adult brains
Source: PeerJ. 2020 Aug 5;8:e9632. doi: 10.7717/peerj.9632 (PMC7414771; doi:10.7717/peerj.9632)
Supplement: Supplementary file 28 [file peerj-08-9632-s004.odt]

| Region Name |  | Cortical Thickness |  | p- value |  | Region Name |  | Cortical Thickness |  | p- value |
| --- | --- | --- | --- | --- | --- | --- | --- | --- | --- | --- |
|  | Val Homozygotes |  | Met Carriers |  |  |  | Val Homozygotes |  | Met Carriers |  |
|  | Median [mm] |  | Mean [mm] |  | S.D. [mm] |  | Median [mm] |  | Mean [mm] |  | S.D. [mm] |  |  |  | Median [mm] |  | Mean [mm] |  | S.D. [mm] |  | Median [mm] |  | Mean [mm] |  | S.D. [mm] |  |
| L_BSTS |  | 3.61 |  | 3.55 |  | 0.33 |  | 3.52 |  | 3.48 |  | 0.38 |  | 0.52 |  | R_BSTS |  | 3.61 |  | 3.55 |  | 0.33 |  | 3.52 |  | 3.48 |  | 0.38 |  | 0.52 |
| L_CACG |  | 2.93 |  | 2.92 |  | 0.26 |  | 3.00 |  | 2.98 |  | 0.30 |  | 0.43 |  | R_CACG |  | 2.93 |  | 2.91 |  | 0.26 |  | 2.99 |  | 2.98 |  | 0.30 |  | 0.43 |
| L_CMFG |  | 3.69 |  | 3.66 |  | 0.30 |  | 3.66 |  | 3.61 |  | 0.34 |  | 0.56 |  | R_CMFG |  | 3.68 |  | 3.66 |  | 0.29 |  | 3.66 |  | 3.60 |  | 0.34 |  | 0.57 |
| L_CU |  | 2.62 |  | 2.61 |  | 0.22 |  | 2.52 |  | 2.57 |  | 0.24 |  | 0.53 |  | R_CU |  | 2.62 |  | 2.61 |  | 0.22 |  | 2.53 |  | 2.57 |  | 0.24 |  | 0.53 |
| L_EC |  | 4.22 |  | 4.14 |  | 0.47 |  | 4.20 |  | 4.16 |  | 0.50 |  | 0.90 |  | R_EC |  | 4.22 |  | 4.14 |  | 0.47 |  | 4.20 |  | 4.16 |  | 0.50 |  | 0.90 |
| L_FG |  | 3.92 |  | 3.87 |  | 0.35 |  | 3.94 |  | 3.84 |  | 0.39 |  | 0.78 |  | R_FG |  | 3.92 |  | 3.88 |  | 0.36 |  | 3.94 |  | 3.84 |  | 0.39 |  | 0.78 |
| L_IPG |  | 3.57 |  | 3.44 |  | 0.29 |  | 3.42 |  | 3.38 |  | 0.31 |  | 0.53 |  | R_IPG |  | 3.57 |  | 3.44 |  | 0.29 |  | 3.42 |  | 3.39 |  | 0.31 |  | 0.54 |
| L_ITG |  | 2.81 |  | 2.79 |  | 0.08 |  | 2.81 |  | 2.83 |  | 0.16 |  | 0.27 |  | R_ITG |  | 2.81 |  | 2.79 |  | 0.09 |  | 2.81 |  | 2.83 |  | 0.16 |  | 0.26 |
| L_ICG |  | 2.57 |  | 2.51 |  | 0.15 |  | 2.50 |  | 2.48 |  | 0.25 |  | 0.60 |  | R_ICG |  | 2.56 |  | 2.50 |  | 0.15 |  | 2.50 |  | 2.47 |  | 0.25 |  | 0.60 |
| L_LOG |  | 2.87 |  | 2.84 |  | 0.22 |  | 2.83 |  | 2.79 |  | 0.24 |  | 0.43 |  | R_LOG |  | 2.87 |  | 2.84 |  | 0.22 |  | 2.83 |  | 2.78 |  | 0.24 |  | 0.43 |
| L_LOFG |  | 3.32 |  | 3.29 |  | 0.19 |  | 3.33 |  | 3.29 |  | 0.25 |  | 0.94 |  | R_LOFG |  | 3.33 |  | 3.30 |  | 0.20 |  | 3.34 |  | 3.29 |  | 0.25 |  | 0.94 |
| L_LG |  | 2.44 |  | 2.43 |  | 0.16 |  | 2.42 |  | 2.42 |  | 0.23 |  | 0.83 |  | R_LG |  | 2.45 |  | 2.44 |  | 0.16 |  | 2.43 |  | 2.42 |  | 0.23 |  | 0.82 |
| L_MOFG |  | 3.20 |  | 3.16 |  | 0.17 |  | 3.18 |  | 3.17 |  | 0.26 |  | 0.91 |  | R_MOFG |  | 3.20 |  | 3.16 |  | 0.18 |  | 3.19 |  | 3.17 |  | 0.26 |  | 0.91 |
| L_MTG |  | 3.34 |  | 3.36 |  | 0.24 |  | 3.43 |  | 3.39 |  | 0.29 |  | 0.65 |  | R_MTG |  | 3.34 |  | 3.36 |  | 0.24 |  | 3.43 |  | 3.39 |  | 0.29 |  | 0.64 |
| L_PHIG |  | 3.53 |  | 3.51 |  | 0.23 |  | 3.43 |  | 3.44 |  | 0.31 |  | 0.36 |  | R_PHIG |  | 3.52 |  | 3.51 |  | 0.23 |  | 3.43 |  | 3.44 |  | 0.31 |  | 0.37 |
| L_PaCG |  | 3.45 |  | 3.38 |  | 0.29 |  | 3.35 |  | 3.30 |  | 0.32 |  | 0.44 |  | R_PaCG |  | 3.45 |  | 3.37 |  | 0.29 |  | 3.34 |  | 3.30 |  | 0.32 |  | 0.44 |
| L_POP |  | 3.70 |  | 3.61 |  | 0.26 |  | 3.62 |  | 3.59 |  | 0.34 |  | 0.86 |  | R_POP |  | 3.70 |  | 3.61 |  | 0.27 |  | 3.62 |  | 3.60 |  | 0.34 |  | 0.86 |
| L_POR |  | 3.08 |  | 3.11 |  | 0.13 |  | 3.18 |  | 3.15 |  | 0.24 |  | 0.46 |  | R_POR |  | 3.08 |  | 3.11 |  | 0.13 |  | 3.18 |  | 3.15 |  | 0.24 |  | 0.46 |
| L_PTR |  | 3.44 |  | 3.36 |  | 0.23 |  | 3.28 |  | 3.26 |  | 0.31 |  | 0.24 |  | R_PTR |  | 3.44 |  | 3.35 |  | 0.23 |  | 3.28 |  | 3.26 |  | 0.31 |  | 0.24 |
| L_PCAL |  | 1.59 |  | 1.54 |  | 0.18 |  | 1.56 |  | 1.61 |  | 0.21 |  | 0.23 |  | R_PCAL |  | 1.59 |  | 1.54 |  | 0.18 |  | 1.56 |  | 1.61 |  | 0.21 |  | 0.23 |
| L_PoCG |  | 2.42 |  | 2.42 |  | 0.16 |  | 2.40 |  | 2.39 |  | 0.17 |  | 0.62 |  | R_PoCG |  | 2.43 |  | 2.42 |  | 0.16 |  | 2.40 |  | 2.39 |  | 0.17 |  | 0.62 |
| L_PCG |  | 2.72 |  | 2.71 |  | 0.18 |  | 2.72 |  | 2.70 |  | 0.26 |  | 0.90 |  | R_PCG |  | 2.72 |  | 2.71 |  | 0.18 |  | 2.72 |  | 2.70 |  | 0.26 |  | 0.90 |
| L_PrCG |  | 2.99 |  | 2.95 |  | 0.20 |  | 2.99 |  | 2.95 |  | 0.25 |  | 0.98 |  | R_PrCG |  | 2.99 |  | 2.95 |  | 0.20 |  | 2.99 |  | 2.96 |  | 0.25 |  | 0.98 |
| L_PCU |  | 3.45 |  | 3.43 |  | 0.26 |  | 3.43 |  | 3.37 |  | 0.34 |  | 0.54 |  | R_PCU |  | 3.45 |  | 3.43 |  | 0.26 |  | 3.43 |  | 3.37 |  | 0.35 |  | 0.53 |
| L_RACG |  | 3.29 |  | 3.35 |  | 0.25 |  | 3.37 |  | 3.38 |  | 0.27 |  | 0.78 |  | R_RACG |  | 3.29 |  | 3.35 |  | 0.25 |  | 3.37 |  | 3.37 |  | 0.27 |  | 0.78 |
| L_RMFG |  | 3.10 |  | 3.05 |  | 0.13 |  | 3.04 |  | 3.01 |  | 0.21 |  | 0.41 |  | R_RMFG |  | 3.10 |  | 3.05 |  | 0.13 |  | 3.05 |  | 3.01 |  | 0.21 |  | 0.41 |
| L_SFG |  | 3.85 |  | 3.79 |  | 0.26 |  | 3.78 |  | 3.74 |  | 0.30 |  | 0.55 |  | R_SFG |  | 3.85 |  | 3.79 |  | 0.26 |  | 3.77 |  | 3.74 |  | 0.30 |  | 0.55 |
| L_SPG |  | 3.15 |  | 3.09 |  | 0.25 |  | 3.08 |  | 3.03 |  | 0.25 |  | 0.49 |  | R_SPG |  | 3.15 |  | 3.09 |  | 0.26 |  | 3.08 |  | 3.03 |  | 0.25 |  | 0.49 |
| L_STG |  | 3.45 |  | 3.49 |  | 0.21 |  | 3.45 |  | 3.44 |  | 0.29 |  | 0.52 |  | R_STG |  | 3.45 |  | 3.49 |  | 0.21 |  | 3.45 |  | 3.44 |  | 0.29 |  | 0.52 |
| L_SMG |  | 3.21 |  | 3.20 |  | 0.20 |  | 3.19 |  | 3.16 |  | 0.23 |  | 0.46 |  | R_SMG |  | 3.21 |  | 3.20 |  | 0.20 |  | 3.18 |  | 3.15 |  | 0.23 |  | 0.46 |
| L_FP |  | 3.23 |  | 3.25 |  | 0.17 |  | 3.10 |  | 3.10 |  | 0.30 |  | 0.03* |  | R_FP |  | 3.23 |  | 3.25 |  | 0.17 |  | 3.10 |  | 3.10 |  | 0.30 |  | 0.03* |
| L_TP |  | 4.73 |  | 4.56 |  | 0.41 |  | 4.49 |  | 4.50 |  | 0.47 |  | 0.63 |  | R_TP |  | 4.72 |  | 4.55 |  | 0.41 |  | 4.49 |  | 4.49 |  | 0.47 |  | 0.63 |
| L_TTG |  | 3.61 |  | 3.60 |  | 0.33 |  | 3.56 |  | 3.50 |  | 0.33 |  | 0.36 |  | R_TTG |  | 3.60 |  | 3.60 |  | 0.33 |  | 3.56 |  | 3.50 |  | 0.33 |  | 0.36 |
| L_IN |  | 4.09 |  | 4.04 |  | 0.29 |  | 3.99 |  | 3.98 |  | 0.35 |  | 0.53 |  | R_IN |  | 4.09 |  | 4.04 |  | 0.29 |  | 3.99 |  | 3.98 |  | 0.35 |  | 0.54 |
